# Strengthening of the intestinal epithelial tight junction by *Bifidobacterium bifidum*

**DOI:** 10.14814/phy2.12327

**Published:** 2015-03-16

**Authors:** Chen-Yu Hsieh, Toshifumi Osaka, Eri Moriyama, Yasuhiro Date, Jun Kikuchi, Satoshi Tsuneda

**Affiliations:** 1 Department of Life Science and Medical Bioscience, Waseda UniversityTokyo, Japan; 2 RIKEN Center for Sustainable Resource ScienceYokohama, Kanagawa, Japan; 3 Graduate School of Medical Life Science, Yokohama City UniversityYokohama, Kanagawa, Japan; 4 Graduate School of Bioagricultural Sciences, Nagoya UniversityNagoya, Aichi, Japan

**Keywords:** ^1^H-NMR, intestinal epithelial permeability, metabonomics, probiotics, tight junctions

## Abstract

Epithelial barrier dysfunction has been implicated as one of the major contributors to the pathogenesis of inflammatory bowel disease. The increase in intestinal permeability allows the translocation of luminal antigens across the intestinal epithelium, leading to the exacerbation of colitis. Thus, therapies targeted at specifically restoring tight junction barrier function are thought to have great potential as an alternative or supplement to immunology-based therapies. In this study, we screened *Bifidobacterium*, *Enterococcus,* and *Lactobacillus* species for beneficial microbes to strengthen the intestinal epithelial barrier, using the human intestinal epithelial cell line (Caco-2) in an in vitro assay. Some *Bifidobacterium* and *Lactobacillus* species prevented epithelial barrier disruption induced by TNF-*α*, as assessed by measuring the transepithelial electrical resistance (TER). Furthermore, live *Bifidobacterium* species promoted wound repair in Caco-2 cell monolayers treated with TNF-*α* for 48 h. Time course ^1^H-NMR-based metabonomics of the culture supernatant revealed markedly enhanced production of acetate after 12 hours of coincubation of *B*. *bifidum* and Caco-2. An increase in TER was observed by the administration of acetate to TNF-*α*-treated Caco-2 monolayers. Interestingly, acetate-induced TER-enhancing effect in the coculture of *B*. *bifidum* and Caco-2 cells depends on the differentiation stage of the intestinal epithelial cells. These results suggest that *Bifidobacterium* species enhance intestinal epithelial barrier function via metabolites such as acetate.

## Introduction

The intestinal mucosa is the dividing line between the internal and external environment, and is continuously exposed to various innocuous and noxious substances in the intestinal lumen. The intestinal epithelium is a single layer of columnar intestinal epithelial cells (IECs) that forms a physical and functional barrier preventing intestinal penetration of unwanted antigens. For the maintenance of epithelial barrier integrity, IECs are tightly bound together by intercellular junctional complexes localized at the apical-lateral membrane and along the lateral membrane (Van Itallie and Anderson 2006). The intercellular junctional complexes consist of the tight junctions (TJ), gap junctions (GJ), adherence junctions (AJ), and desmosome (Tsukita and Furuse 2002; Groschwitz and Hogan 2009; Marchiand et al. 2010). In particular, the apical junctional complex, encompassing the TJ (composed of claudins and occludin) and AJ (containing E-cadherin), plays a crucial role in regulating epithelial barrier functions (Laukoetter et al. 2006).

Intestinal permeability reflects the state of the epithelial TJ (Van Itallie and Anderson 2006). The TJ seals the intercellular space between adjacent epithelial cells, and is responsible for regulating selective paracellular transport of ions and solutes (e.g., nutrients) and preventing the translocation of microorganisms (both commensals and pathogens) and antigens (including bacterial toxins and food components) across the epithelium (Tsukita and Furuse 2002; Balda and Matter 2008). However, some pathogens (e.g., *Salmonella* spp.) impair or subvert the intestinal epithelial TJ barrier, and subsequently cause acute inflammation (O'Hara and Buret 2008). Moreover, chronic inflammation is often associated with defective intestinal epithelial TJ barrier function that allows luminal antigens to stimulate underlying immune cells in various diseases (including celiac disease, Crohn's disease, diabetes, and food allergy) (Vogelsang et al. 1998; Suenaert et al. 2002; Ventura et al. 2006).

Intestinal permeability is affected by multiple factors including cytokines (notably TNF-*α* and IFN-*γ*), epithelial apoptosis, and exogenous factors (including alcohol, high-fat diet, nonsteroidal antiinflammatory drugs) (Ferrier et al. 2006; Ma et al. 2006, 2008; Oshima et al. 2008; Groschwitz and Hogan 2009). Cytokine-mediated dysregulation of intestinal barrier function has been implicated as an etiological factor for inflammatory bowel disease (IBD) and numerous autoimmune diseases (Visser et al. 2009; Maloy and Powrie 2011). Especially in Crohn's disease, where TNF-*α* is thought to be a critical determinant of the predisposition to intestinal inflammation and a defective epithelial TJ barrier because treatment with anti-TNF-*α* antibodies improved impaired intestinal barrier function in Crohn's disease patients (Targan et al. 1997; Van Deventer 1997; Noth et al. 2012). TNF-*α* is known to be not only a proinflammatory cytokine that activates the endogenous inflammatory cascade but also a highly potent inducer of epithelial TJ disruption (Ma et al. 2005; Ye et al. 2006; He et al. 2012). The TNF-*α*-induced increase in intestinal TJ permeability is associated with downregulation of zonula occludens 1 (ZO-1) protein expression and an increase in myosin light chain kinase (MLCK)-mediated opening of the intestinal epithelial TJ barrier in a NF-*κ*B-dependent manner.

*Bifidobacterium* species, *Enterococcus* species, *Escherichia coli*, *Lactobacillus* species, *Streptococcus* species, and *Lactococcus lactis*, most of which were originally isolated from healthy humans, have been used as probiotics to confer benefits to the host (De Vrese and Schrezenmeir 2008). Some of these probiotics have been shown to promote intestinal epithelial barrier integrity via modulation of the TJ in a strain-dependent manner (Miyauchi et al. 2012; Sultana et al. 2013). Numerous previous studies showed that pretreatment of human intestinal epithelial cell lines (specifically Caco-2, HT-29, and T-84) or animal models of colitis with probiotic bacteria confers protective effects against TJ barrier impairment induced by various factors, including pathogen infection, proinflammatory cytokines, and oxidative stress (Resta-Lenert and Barrett 2003, 2006; Ewaschuk et al. 2008; Ohland and MacNaughton 2010). These TJ-strengthening probiotics upregulated the expression of TJ proteins (notably occludin and ZO-1) or downregulated the expression of the pore-forming protein claudin-2, suggesting that probiotics directly regulate TJ barrier function at the gene expression level (Resta-Lenert and Barrett 2003; Ewaschuk et al. 2008). Furthermore, probiotics modulated various protein kinase signaling pathways, which enhanced phosphorylation of TJ proteins, which can either promote TJ formation and barrier function or alternatively promote TJ protein redistribution and complex destabilization (Resta-Lenert and Barrett 2003; Zyrek et al. 2007). However, the bacterial components and metabolites responsible for the TJ-strengthening effects have not yet been fully clarified.

Although protective effects on TJ integrity against harmful stimuli by pretreatment with probiotics have been well characterized as described above, there are only a limited number of studies focusing on the therapeutic effects of probiotics on impaired epithelial barrier function. In this study, we screened for beneficial microbes that promote the restoration of the intestinal epithelial barrier function under inflammatory conditions from intestinal microorganisms including *Bifidobacterium*, *Enterococcus,* and *Lactobacillus* species originating from human feces. The aim of this study was to investigate the mechanisms underlying the therapeutic effects of the screened bacteria. Furthermore, we tried to identify candidate metabolites responsible for the TJ-strengthening effects using ^1^H-NMR-based metabonomics.

## Materials and Methods

### Caco-2 cell culture

The human colonic adenocarcinoma epithelial cell line Caco-2 was obtained from Riken Cell Bank (Ibaraki, Japan) and kept in a humidified incubator at 37°C with 5% CO_2_. Caco-2 cells were maintained in Dulbecco's Modified Eagle Medium (DMEM)-High glucose (Wako, Osaka, Japan) supplemented with 10% heat-inactivated (30 min, 56°C) fetal bovine serum (Biowest, Nuaillé, France), 100 U/mL penicillin, 100 *μ*g/mL streptomycin, and 1% nonessential amino acids (NEAA) (Wako). In this study, Caco-2 cells were used between passages 25 and 40. Cells cultivated to 80% confluence were seeded on 12-well Transwell® inserts (1.12 cm^2^ polycarbonate membrane with 0.4 *μ*m pore size; Corning Life Sciences, Corning, NY) at a density of 5 × 10^4^ cells per well. The culture medium was changed every 2 days until 20 days later when full polarization of the Caco-2 cell monolayer was achieved. The integrity of the Caco-2 cell monolayer was evaluated by measuring the transepithelial electrical resistance (TER) using a milicell-ERS (Millipore, Billerica, MA). The TER values measured in the experiments were summarized as table form below each figure, and all the figures were expressed as the relative TER value compared to the value before experimental treatment.

### Bacterial isolates and sample preparation

Thirty bacterial isolates from human feces were used in this study (Table1). Each bacterial isolate was identified based on a nearly full 16S rRNA sequence, which was deposited in DNA Data Bank of Japan (DDBJ). All bacterial strains were cultured in Difco Lactobacilli MRS (de Man-Rogosa Sharpe) broth (Becton Dickinson, Sparks, MD) at 37°C under anaerobic conditions. Heat-killed bacteria were prepared by heating bacteria at 95°C for 10 min.

**Table 1:** List of bacterial isolates used in this study

Strain	Accession number
Lactobacillus	
*L*. *salivarius* WU 30	AB932525
*L*. *fermentum* WU 30	AB932548
*L*. *fermentum* WU 33	AB932537
*L*. *gasseri* WU 04	AB932527
*L*. *gasseri* WU 06	AB932530
*L*. *rhamnosus* WU 08	AB932536
*L*. *gasseri* WU 11	AB932519
*L*. *pantheris* WU 61	AB932532
*L*. *pantheris* WU 21	AB932522
*L*. *rhamnosus* WU 07	AB932535
*L*. *rhamnosus* WU 09	AB932547
*L*. *rhamnosus* WU 12	AB932520
*L*. *rhamnosus* WU 14	AB932521
*L*. *salivarius* WU 57	AB932528
*L*. *salivarius* WU 60	AB932531
*L*. *salivarius* WU 63	AB932533
Enterococcus	
*E*. *cecorum* WU 65	AB932534
*E*. *faecium* WU 31	AB932549
*E*. *avium* WU 58	AB932529
*E*. *avium* WU 22	AB932523
*E*. *avium* WU 76	AB932546
*E*. *raffinosus* WU 27	AB932524
Bifidobacterium	
*B*. *bifidum* WU 11	AB932538
*B*. *bifidum* WU 12	AB932539
*B*. *bifidum* WU 20	AB932541
*B*. *bifidum* WU 57	AB932544
*B*. *longum* WU 16	AB932540
*B*. *longum* WU 35	AB932543
*B*. *adolescentis* WU 22	AB932542
*B*. *pseudocatenulatum* WU 06	AB932550

### Evaluation of the TJ-barrier-strengthening ability of bacterial isolates

To screen the TJ-barrier-strengthening bacteria, polarized Caco-2 cell monolayers were exposed to bacterial isolates. On day 18 of cultivation, both apical and basal compartments of Caco-2 cell monolayers were washed twice with prewarmed PBS and then fresh DMEM without antibiotics added to both compartments of the Transwell® cell culture system. On day 20, the TER of each well was measured using a milicell-ERS. Caco-2 cell monolayers with >650 Ω cm^2^ of TER were used for further experiments. After the measurement of TER, the apical compartment of Caco-2 cell monolayers was washed with prewarmed PBS, and then the bacterial suspensions were exposed to the apical side at a multiplicity of infection (MOI, ratio of bacteria number to epithelial cell number) of 1 in a humidified incubator at 37°C with 5% CO_2_. Culture medium alone was used as a negative control. After 24 h incubation, the TER value was measured to assess epithelial TJ permeability. All experiments were performed in triplicate.

### Evaluation of preventive effect of bacterial isolates on TNF-α-induced impairment of TJ permeability

In the prevention screening, Caco-2 cell monolayers were washed with prewarmed PBS and then bacteria (MOI of 1) added to the apical compartment one hour before TNF-*α* treatment. Next, the Caco-2 cell monolayers were treated with TNF-*α* (20 ng/mL) from the basolateral compartment to induce TJ dysfunction and incubated for a further 23 h. The TER value was measured to assess epithelial barrier function after a total of 24 h incubation. The TNF-*α* treated group without bacteria was used as a negative control. The prevention effect was estimated by the change in TER compared with the value before bacteria was added. All experiments except for *B*. *bifidum* WU57 were performed in triplicate. The assay of *B*. *bifidum* WU57 was performed in duplicate.

### Evaluation of restorative effect of bacterial isolates on TNF-α-induced impairment of TJ permeability

Fully polarized Caco-2 cell monolayers were washed with prewarmed PBS then antibiotic-free DMEM containing TNF-*α* was added to the basolateral compartment. After 48 h incubation, the Caco-2 cell monolayers were washed on both the apical and basolateral sides with prewarmed PBS. Subsequently, the apical compartment of the Caco-2 cell monolayers was exposed to the bacterial suspensions at a MOI of 1. After 24 h incubation, the TER value was measured to assess the restoration of TJ function. All experiments were performed in triplicate.

### RNA isolation and Gene expression analysis

Total RNA was extracted using the RNeasy mini kit (Qiagen, Tokyo, Japan) according to the manufacturer's instructions, and the RNA concentration determined by absorbance at 260/280 nm using a spectrophotometer (nano-Drop ND-1000; Thermo Scientific, Wilmington, DE). For mRNA expression analysis, cDNA was prepared from 1500 to 2000 ng of total RNA using the High-Capacity cDNA reverse transcription kit (Applied Biosystems, Carlsbad, CA) according to the manufacturer's instructions. The reverse transcription reactions were performed in a thermo cycler (iCycler, Bio-Rad, Hercules, CA) at 25°C for 10 min, 37°C for 120 min, and 85°C for 5 min. Real-Time PCR was performed with a StepOnePlus™ Real-time PCR System (Applied Biosystems) using the TaqMan® Gene Expression Assays No. Hs00268480_m1 (ZO-1), Hs00170162_m1 (occludin), Hs00221623_m1 (claudin-1), Hs00170423_m1 (E-cadherin), and Hs99999905_m1 (GAPDH) with TaqMan gene expression master mix according to the manufacturer's instructions (Applied Biosystems). The time-dependent TJ gene expression analysis during the coculture of *B*. *bifidum* WU12 and Caco-2 cells were repeated two independent times, each performed in duplicate or triplicate. The endpoint TJ gene expression analysis of Caco-2 cells treated with *B*. *bifidum* WU12 or acetate for 24 h were repeated three independent times, each performed in triplicate.

### Characterization of metabolites in culture supernatants using ^1^H-NMR spectroscopy

Supernatants of *B*. *bifidum* monocultures and the coculture of TNF-*α*-treated Caco-2 cells and *B*. *bifidum* were collected at 6, 12, and 24 h. DMEM and the culture supernatants of Caco-2 cell monolayers treated with TNF-*α* for 48 h were also prepared. The metabolic profile of the culture supernatant was analyzed using a NMR spectrometer. The NMR samples were prepared by mixing with 10 mmol/L sodium 2,2-dimethyl-2-silapentane-5-sulfonate (DSS) dissolved in deuterium oxide (D_2_O) and then transferred into 5 mm NMR tubes. The spectra of these supernatant samples were obtained on a Bruker DRU-700 spectrometer equipped with an inverse (proton coils closest to the sample) gradient 5 mm cryogenically cooled ^1^H/^13^C/^15^N probe (Bruker Biospin, Rheinstetten, Germany), operating at 700.15 MHz for protons. The NMR measurement and data analysis of Carr–Purcell–Meiboom–Gill (CPMG) pulse sequences was performed as per previous studies (Date et al. 2012). The spectra acquired at 298 K, and 32,768 data point with a spectral width of 12,500 Hz were collected into 32 transients and 16 dummy scans. The NMR spectra were processed using TopSpin 3.1 software (Bruker Biospin) and assigned using the SpinAssign programs at the PRIMe web site (http://prime.psc.riken.jp/) (Chikayama et al. 2010). The CPMG spectra data were reduced by subdividing spectra into sequential 0.04 ppm-designated regions between ^1^H chemical shifts of −0.5 to 9.0 ppm. After exclusion of water resonance (4.7–5.0 ppm), each region was integrated and normalized to the total of DSS integral regions. The normalized spectra data were statistically evaluated by Principle Component Analysis (PCA) using the ‘R (2.15.2)’ software.

### Determination of acetate, formate, and lactate in culture supernatants

Supernatant samples were treated with an equal volume of acetonitrile (Wako) at room temperature to precipitate proteins, and then centrifuged for 10 min at 20,000 × *g* at room temperature. The supernatant was collected by filtration through a 0.2 *μ*m Advantec® disposable membrane filter unit (Toyo Roshi, Tokyo, Japan) and then stored at −80°C until analysis. Samples were diluted 100-fold in ddH_2_O on the eve of analysis. Acetate, formate, and lactate content in culture supernatants was determined by ICS 2100 ion chromatography (Dionex Instruments, Sunnyvale, CA) with a Dionex IonPac AS-19 column and a 1–40 mmol/L KOH gradient mobile phase.

## Results

### Exploration of microorganisms strengthening tight junction barrier

**Figure 1:**
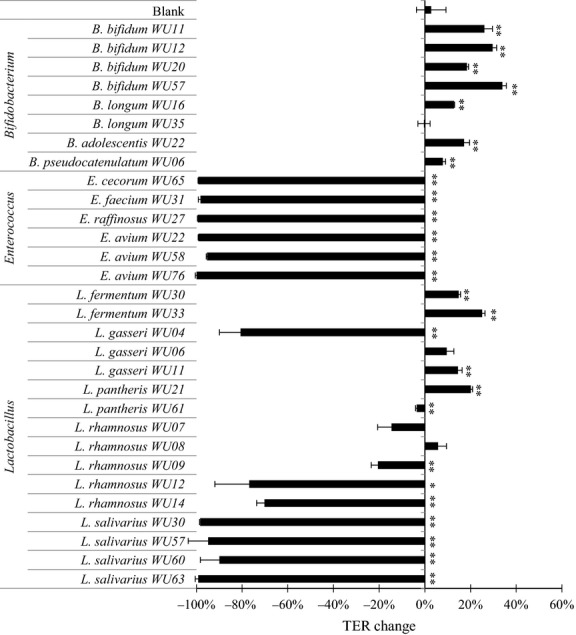
Screening of bacterial isolates for TJ-barrier-strengthening effects in polarized Caco-2 monolayers at MOI = 1. All the bacteria isolates were incubated with Caco-2 monolayers for 24 h. The average of actual TER values of Caco-2 monolayer used in this experiment was 1057 ± 98 Ω cm^2^. Experiments were carried out in triplicate, and data represent the means of relative changes in TER ± SD. **P < 0.01, *P < 0.05 compared with the blank (media alone) control group by *t*-test.

After the polarized Caco-2 cell monolayers were incubated with each bacterial isolate or medium alone for 24 h, the intestinal barrier function was determined by measurement of the TER (Fig.1 and Table2). All *Enterococcus* isolates (including *E*. *faecium*, *E*. *avium*, *E*. *cecorum,* and *E*. *raffinosus*) induced an appreciable decrease in TER. Some *Lactobacillus* species (*L*. *gasseri*, *L*. *rhamnosus*, and *L*. *salivarius*) also induced a significant decrease in TER. In contrast, seven *Bifidobacterium* isolates and six *Lactobacillus* isolates induced a 5.7–33.9% increase in TER. Therefore, we examined the preventive effects of these screened isolates (7 strains of *Bifidobacterium* and 6 strains of *Lactobacillus*) on TNF-*α*-induced impairment of TJ permeability.

**Table 2:** The TER value measured at the screening of bacterial isolates for TJ-barrier-strengthening effects in polarized Caco-2 monolayers

Genus	Species	Strain	0 h		24 h coincubation	
			TER (Ω × cm^2^)	±SD	TER (Ω × cm^2^)	±SD
–	–	Blank	1085.1	53.7	1115.3	90.5
*Lactobacillus*	*fermentum*	WU30	1194.5	16.7	1371.8	13.8
		WU33	927.9	32.6	1160.9	38.1
	*gasseri*	WU04	919.3	37.5	176.4	81.5
		WU06	1056.3	24.8	1157.1	38.7
		WU11	1074.6	57.8	1229.9	49.8
	*pantheris*	WU21	1069.0	23.4	1283.7	35.6
		WU61	1098.9	31.4	1059.3	24.9
	*rhamnosus*	WU07	1109.4	22.7	948.5	87.2
		WU08	1095.9	33.4	1158.6	44.4
		WU09	1138.9	31.1	904.4	22.7
		WU12	1171.7	18.9	272.3	177.9
		WU14	1150.8	24.4	342.5	35.3
	*salivarius*	WU30	1160.1	67.2	19.2	2.3
		WU57	1081.7	3.4	55.8	94.7
		WU60	1089.6	43.5	109.2	89.4
		WU63	1159.8	71.9	9.5	15.5
*Enterococcus*	*avium*	WU22	1050.4	51.5	9.9	2.6
		WU58	1092.9	30.6	51.0	3.0
		WU76	935.8	45.1	0.9	5.6
	*cecorum*	WU65	1100.4	31.4	9.9	2.3
	*faecium*	WU31	1162.0	27.2	20.7	10.8
	*raffinosus*	WU27	1135.5	71.2	6.5	1.7
*Bifidobacterium*	*adolescentis*	WU22	959.7	15.2	1123.9	25.6
	*bifidum*	WU11	911.9	60.8	1148.6	54.7
		WU12	900.3	74.1	1166.5	82.0
		WU20	1085.1	32.1	1284.5	30.4
		WU57	873.8	15.5	1170.2	29.8
	*longum*	WU16	1029.1	19.5	1160.5	23.6
		WU35	1033.9	21.5	1030.2	38.7
	*pseudocatenulatum*	WU06	923.8	100.5	997.4	114.5

### Evaluation of preventive effect of screened bacteria on TNF-α-induced impairment of tight junction function

**Figure 2:**
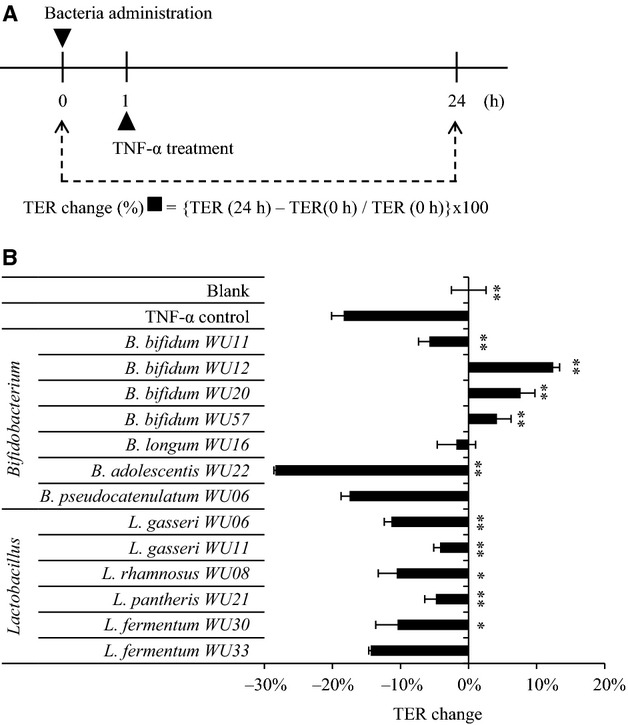
The preventive effect against TNF-*α*-induced TJ barrier impairment. (A) Schedule of Caco-2 monolayer treatment and TER analysis. The bacterial suspensions were added to the apical side of Caco-2 monolayers an hour before TNF-*α* stimulation. After 1 h, Caco-2 monolayers were stimulated on the basolateral side with TNF-*α*, and then incubated for 24 h. (B) The preventive effect of bacterial isolates with barrier-strengthening ability at MOI = 1. The average of actual TER values of Caco-2 monolayer used in this experiment was 976 ± 166 Ω cm^2^. Experiments were carried out in triplicate, and data represent the means of relative changes of TER ± SD. **P < 0.01, *P < 0.05 compared with the TNF-*α* control group by *t*-test.

TNF-*α* treatment induced a marked decrease in TER of the Caco-2 cell monolayers. Pretreatment with *Lactobacillus* strains did not attenuate the TNF-*α*-induced decrease in TER (Fig.2 and Table3). In contrast, four of the seven *Bifidobacteria* strains prevented the TNF-*α*-induced drop in TER. Interestingly, exposure to three *Bifidobacteria* strains (*B*. *bifidum* WU12, WU20, and WU57) led to a reproducible and significant 4.2–12.4% increase in TER (*P* < 0.01). Additionally, one *Bifidobacteria* strain (*B*. *bifidum* WU11) and two *Lactobacillus* strains (*L*. *gasseri* WU11 and *L*. *pantheris* WU21) demonstrated partial suppression of the TNF-*α*-induced TER drop, (−4.2 to −5.8% change).

**Table 3:** The TER value measured at the screening of preventive effect against TNF-*α*-induced TJ barrier impairment

Genus	Species	Strain	0 h		24 h coincubation	
			TER (Ω × cm^2^)	±SD	TER (Ω × cm^2^)	±SD
–	–	Blank	979.1	79.5	978.3	63.1
–	–	TNF-*α* control	948.5	117.2	774.1	92.9
*Lactobacillus*	*fermentum*	WU30	1262.1	32.4	1129.5	11.4
		WU33	996.6	35.1	853.3	27.3
	*gasseri*	WU06	1215.8	67.3	1078.4	67.6
		WU11	846.2	35.9	810.7	32.6
	*pantheris*	WU21	880.5	19.8	837.9	9.8
	*rhamnosus*	WU08	1235.2	36.4	1104.1	10.6
*Bifidobacterium*	*adolescentis*	WU22	1031.7	49.1	739.0	34.1
	*bifidum*	WU11	858.1	67.3	808.1	51.0
		WU12	751.0	34.7	844.3	35.1
		WU20	775.2	16.1	834.2	1.3
		WU57	794.6	1.6	827.7	18.2
	*longum*	WU16	1043.7	47.7	1024.2	28.4
	*pseudocatenulatum*	WU06	958.2	21.4	790.5	10.8

### Evaluation of bacteria-induced restoration of epithelial function

**Figure 3:**
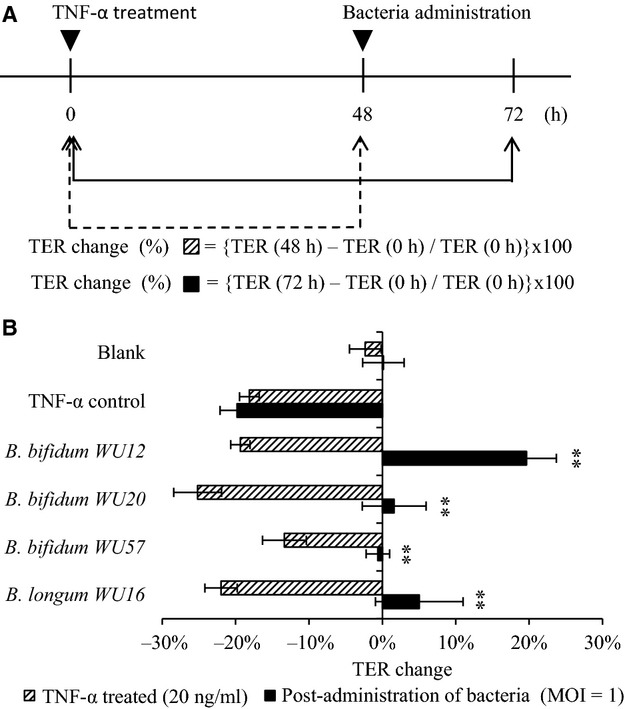
Repair of TNF-*α*-induced barrier dysfunction by administration of bacterial isolates post TNF-*α* stimulation. (A) The schedule of TNF-*α* and bacterial treatment of Caco-2 monolayers. Caco-2 monolayers were stimulated on the basolateral side with TNF-*α*, and then incubated for 48 h. After 48 h, the bacterial suspension were added into the apical side, and coincubated for 24 h. (B) Repair effect of four Bifidobacterial species on the TJ function of Caco-2 cell monolayer damaged by basolateral TNF-*α* stimulation. The average of actual TER values of Caco-2 monolayer used in this experiment was 879 ± 147 Ω cm^2^. Experiments were carried out in triplicate, and data represent the means of relative changes in TER ± SD. **P < 0.01 compared with the TNF-*α* treated TER of the same group by *t*-test.

In this study, the addition of TNF-*α* (20 ng/mL) to the basolateral compartment produced an approximately 15–20% drop in Caco-2 TER for 48 h. TER of TNF-*α*-treated Caco-2 cell monolayers did not increase after changing to fresh medium without TNF-*α*, indicating no spontaneous recovery of the Caco-2 monolayer TJ function. Based on the above results, it was hypothesized that the four *Bifidobacteria* strains (*B*. *bifidum* strain WU12, WU20, WU57, and *B*. *longum* strain WU16) which exhibited protective effects against TNF-*α* induced damage could also be involved in the restoration of intestinal epithelial TJ function. Thus, we examined whether *Bifidobacteria* strains can restore the TJ function of Caco-2 cell monolayers damaged by the treatment of TNF-*α*. As a result, all tested *Bifidobacterium* (3 strains of *B*. *bifidum*, 1 strain of *B*. *longum*) facilitated the restoration of the Caco-2 TJ function (Fig.3 and Table4). To elucidate the mechanism of *Bifidobacteria*-induced epithelial restoration, *B*. *bifidum* strain WU12 was chosen for further study.

**Table 4:** The TER value measured at the repairing of TNF-*α*-induced barrier dysfunction by administration of bacterial isolates post TNF-*α* stimulation

Genus	Species	Strain	Before treatment		TNF-*α* treated		24 h coincubation	
			TER (Ω × cm2)	±SD	TER (Ω × cm2)	±SD	TER (Ω × cm2)	±SD
–	–	Blank	696.8	14.9	680.8	27.4	697.9	30.2
–	–	TNF-*α* control	1050.6	14.7	860.2	20.7	842.2	12.7
*Bifidobacterium*	*longum*	WU16	1026.9	71.2	800.2	37.8	1077.6	83.0
	*bifidum*	WU12	884.6	66.6	712.9	41.8	1058.2	84.1
		WU20	903.3	40.0	675.5	32.6	918.6	73.4
		WU57	709.1	36.5	613.9	20.2	705.0	44.0

### Characterization of the factor responsible for *B*. *bifidum*-induced restoration of epithelial function

**Figure 4:**
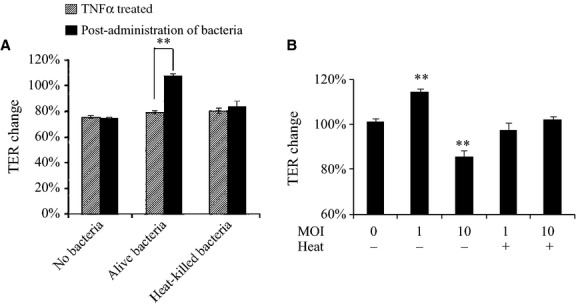
Characterization of the factor responsible for *B*. *bifidum*-induced TJ restoration. (A) Effects of the heat treatment (95°C for 10 min) of bacterial cells on *B*. *bifidum* WU12-induced epithelial TJ restoration capacity. The average of actual TER values of Caco-2 monolayer before basolateral TNF-*α* stimulation used in this experiment was 894 ± 99 Ω cm^2^. Experiments were carried out in triplicate, and data represent the means of relative changes in TER ± SD before and after administration of *B*. *bifidum* WU12. Statistical differences between before and after administration of *B*. *bifidum* WU12 in each experimental group were calculated by *t*-test (**P < 0.01). (B) The cytotoxic effect of MOI on cocultivation of Caco-2 monolayers with alive or heat-killed *B*. *bifidum* WU12. The average of actual TER values of Caco-2 monolayer used in this experiment was 753 ± 91 Ω cm^2^. Experiments were carried out in triplicate, and data represent the means of relative changes of TER ± SD. Statistical differences before and after administration of alive or heat-killed *B*. *bifidum* WU12 were calculated by *t*-test (**P < 0.01).

We determined whether the bacterial components or secreted metabolites were responsible for the epithelial TJ restoration induced by *B*. *bifidum* strain WU12. Heat-killed *B*. *Bifidum* WU12 clearly had a diminished a repair capacity (Fig.4A and Table5A). Interestingly, only intact cells of *B*. *bifidum* strain WU12 resulted in a pronounced increase in TER of the Caco-2 cell monolayers, indicating a high repair capacity on the epithelial TJ. Thus, the interaction between live *Bifidobacterium* and Caco-2 cells are a prerequisite for *Bifidobacteria*-induced epithelial restoration. However, high-dose administration of intact bacterial cells at a MOI of 10 resulted in a significant decrease in TER of TNF-*α*-untreated Caco-2 cell monolayers (Fig.4B and Table5B). This might be due to the acceleration of medium acidification, as suggested by the medium turning yellow during the coculture of Caco-2 cells and live *Bifidobacterium*.

**Table 5:** The TER value measured at the (A) The effects of MOI on cocultivation of Caco-2 monolayers with live/heat inactive *B*. *bifidum* WU12, (B) characterization of *B*. *bifidum* WU12-induced epithelial TJ restoration with live/heat-killed bacteria

(A)				
Sample	TNF-*α* treated		Postadministration of bacteria	
	TER (Ω × cm^2^)	±SD	TER (Ω × cm^2^)	±SD
No bacteria	734.5	13.4	727.1	18.8
Alive bacteria (MOI=1)	652.0	11.7	888.0	12.1
Heat-killed bacteria (MOI=1)	709.1	102.1	739.4	90.0

(B)				
Sample	0 h		24 h coincubation	
	TER (Ω × cm^2^)	±SD	TER (Ω × cm^2^)	±SD
No bacteria	867.4	71.2	879.0	78.1
Alive bacteria (MOI=1)	692.7	75.9	791.3	89.5
Alive bacteria (MOI=10)	655.0	42.3	558.7	19.7
Heat-killed bacteria (MOI=1)	739.8	24.2	721.5	17.0
Heat-killed bacteria (MOI=10)	807.7	4.7	824.1	8.5

### Modulation of the mRNA expression of tight junction proteins by *B*. *bifidum*

**Figure 5:**
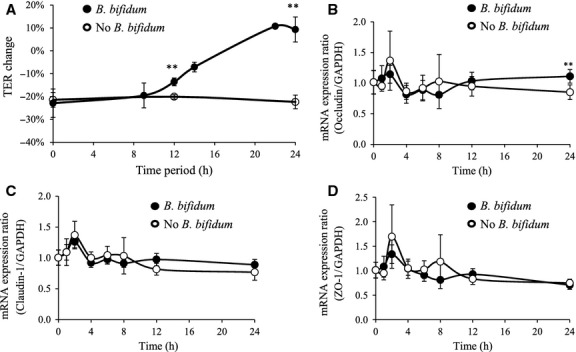
The kinetics of TER and TJ-related mRNA expression during *B*. *bifidum* WU12-induced Caco-2 monolayer restitution. (A) *B*. *bifidum* WU12-induced Caco-2 monolayer restitution over time as estimated by TER. Caco-2 monolayers incubated without *B*. *bifidum* WU12 were used as the negative control. The average of actual TER values of Caco-2 monolayer before basolateral TNF-*α* stimulation used in this experiment was 822 ± 86 Ω cm^2^. Experiments were carried out in triplicate, and data represent the means of relative changes of TER ± SD. (B–D) Temporal changes of mRNA expression changes in TJ protein genes (Claudin-1, Occludin, ZO-1) during Caco-2 monolayer restitution induced by *B*. *bifidum* WU12. The time course of mRNA expression of nontreated group (w/o *B*. *bifidum*) was used as the negative control. Experiments were carried out in triplicate, and data represent the means of relative changes in ± SD. Statistical differences were calculated by *t*-test, comparing conditions with the negative control group at the same time point (**P < 0.01).

The kinetics of TER change during *B*. *bifidum*-induced epithelial restoration is shown in Figure5A and Table6. A rapid increase in TER was observed after 12 h of coincubation which reached 9.3% at 24 h. Quantitative RT-PCR (qRT-PCR) analysis was performed to characterize the changes in gene expression of tight junction proteins during *B*. *bifidum*-induced epithelial restoration. Although the expression dynamics of the TJ protein genes during the coincubation period were not correlated with the change in TER in Caco-2 cells incubated with *B*. *bifidum*, the administration of *B*. *bifidum* significantly enhanced the occludin mRNA expression after 24 h incubation (Fig.5B, C, and D).

**Table 6:** The TER value measured during the *B*. *bifidum* WU12-induced Caco-2 monolayer restitution in 24 h

Sample	*B*. *bifidum*		NO *B*. *bifidum*	
	TER (Ω × cm^2^)	±SD	TER (Ω × cm^2^)	±SD
Naïve cell	890.0	124.7	762.1	35.8
0 h (TNF-*α* treated)	682.9	89.1	598.1	20.0
9 h	727.6	71.1		
12 h	782.5	111.3	598.5	18.8
14 h	706.3	17.4		
22 h	910.9	20.1		
24 h	869.9	14.2	596.6	24.8

### Time-course characterization of the coculture supernatant

**Figure 6:**
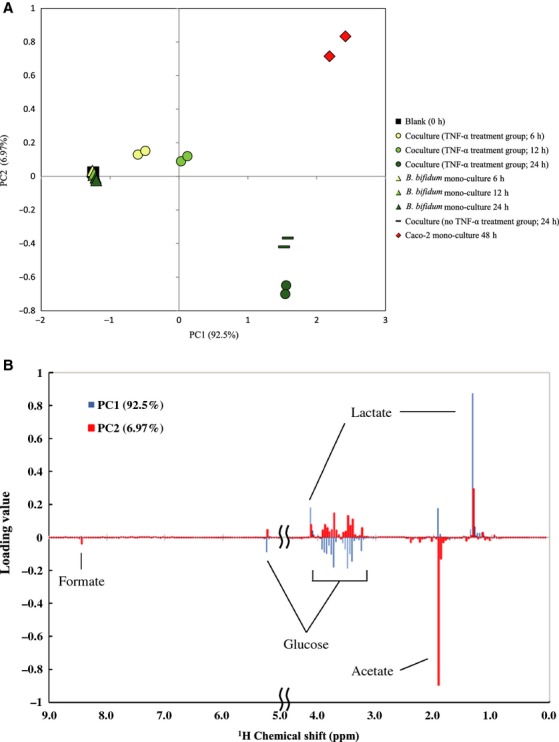
Principle component analysis (PCA) score plots and loading plots of the metabolic profile affected by *B*. *bifidum* WU12. (A) PCA score plot was computed from blank (square), Caco-2 mono-culture 48 h (with TNF-*α*; diamond), time-dependent change of the coculture group (TNF-*α* treated; circle), time-dependent change in *B*. *bifidum* WU 12 mono-culture group (triangle), *B*. *bifidum* WU12 coculture control (dash). (B) PCA loading plot derived from the information on the PCA score plots. Experiments were carried out in duplicate.

Based on the above results, it was hypothesized that metabolites produced by *B*. *bifidum* strain WU12 play a crucial role in the process of *Bifidobacteria*-induced epithelial restoration. To characterize the metabolic dynamics during the epithelial restoration, time-course metabolic profiles of the apical side supernatant were determined using a ^1^H-NMR-based metabonomics approach. The principal component analysis (PCA) of the ^1^H NMR chemical shift data revealed clear differences in metabolite profiles between the coculture groups, the monoculture of *B*. *bifidum*, and the monoculture of Caco-2 cell monolayers (Fig.6). Contributions of the first two components (PC1 and PC2) were 92.5% and 6.97%, respectively (Fig.6A). The loading plot analysis showed that the major metabolites contributing to the separation of each component were lactate and glucose in PC1 and acetate and formate in PC2 (Fig.6B). In the metabolite profile of *B*. *bifidum* alone group, there was only a minute amount of acetate production detected during cultivation. In the Caco-2 monoculture treated with TNF-*α* for 48 h, glucose was consumed and a large amount of lactate produced. In contrast, the coculture of *B*. *bifidum* and Caco-2 notably enhanced the production of acetate after 12 h of the incubation. Furthermore, formate was also produced in the coculture of *B*. *bifidum* and Caco-2.

### Evaluation of the production of acetate, formate, and lactate in the coculture supernatant of Bifidobacterium isolates and Caco-2 cells

**Figure 7:**
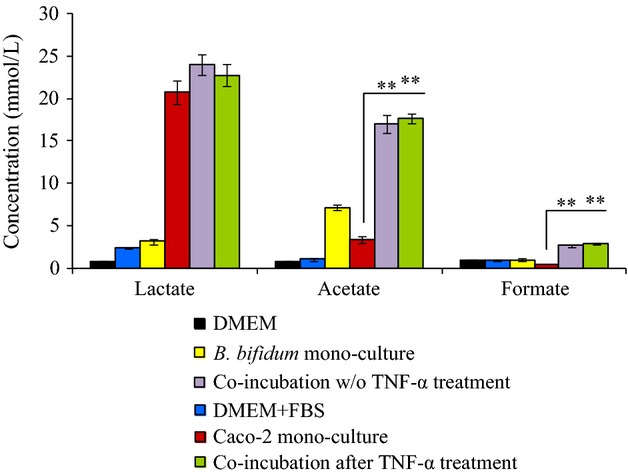
Increased production of acetate and formate by the cocultures of Caco-2 monolayer and *B*. *bifidum* WU. Acetate, formate, and lactate concentrations in the culture supernatant after 24 h incubation were determined by ion chromatography. Experiments were carried out in triplicate, and data represent the means ± SD. Statistical differences were calculated by *t*-test (**P < 0.01).

**Figure 8:**
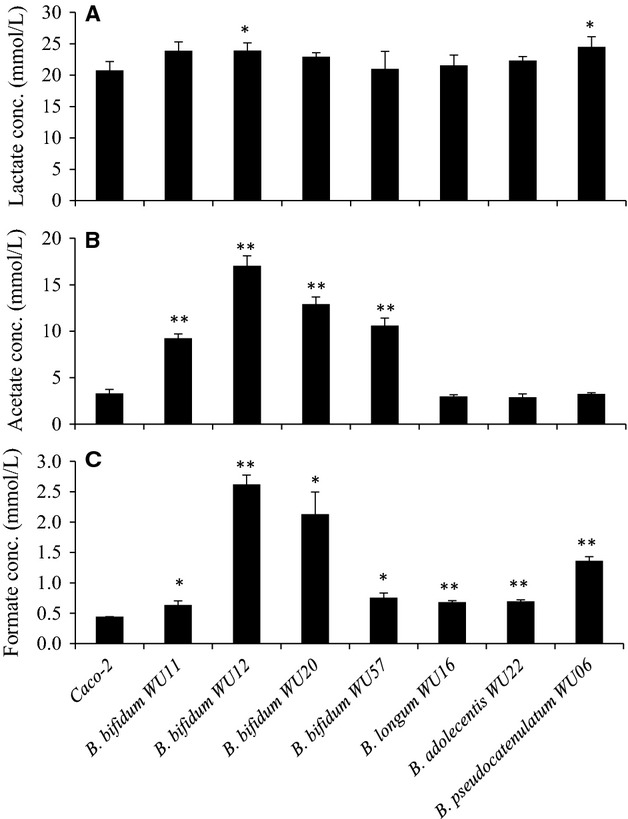
Production of acetate and formate in the coculture supernatant varied in a strain-dependent manner. (A–C) Acetate, formate, and lactate concentrations in the supernatant after 24 h cocultures of Caco-2 monolayer and seven different Bifidobacterial strains were determined by ion chromatography. Experiments were carried out in triplicate, and data represent the means ± SD. Statistical differences were calculated by *t*-test (**P < 0.01, *P < 0.05).

We conducted ion chromatography analysis to determine the production of acetate, formate and lactate in the supernatant during coculture of Caco-2 and *B*. *bifidum* WU12, with and without TNF-*α*, and by *B*. *bifidum* alone (Fig.7). Interestingly, the production of acetate and formate was amplified by the coculture of *B*. *bifidum* WU12 and Caco-2 monolayers. Acetate production in the coculture group increased 1.8 times compared with that of the monoculture of the *B*. *bifidum* strain WU12. Furthermore, we determined whether the production of acetate, formate, and lactate in the coculture supernatant differed between the seven *Bifidobacterium* strains, which fortified the epithelial TJ barrier of naive polarized Caco-2 cells (Fig.1). Analysis of the culture supernatant revealed that the production of lactate was mainly derived from cell metabolism of Caco-2 cells as there were no differences between the monoculture of Caco-2 and the coculture of Caco-2 and *Bifidobacterium* (Fig.8). In contrast, the production of acetate and formate varied among *Bifidobacterium* species. Notably, *B*. *bifidum* produced higher levels of acetate and formate in comparison with other species (*B*. *adolescentis, B*. *longum,* and *B*. *pseudocatenulatum*) (Fig.8).

### Effects of acetate and formate on TJ barrier function in Caco-2 monolayers

**Figure 9:**
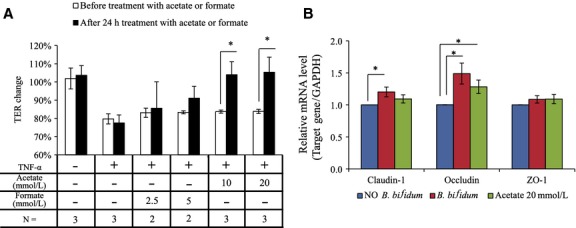
Acetate induced the TJ barrier restorative capacity. (A) Effects of acetate and formate on the TJ barrier restoration. Caco-2 monolayers were stimulated on the basolateral side with TNF-*α*, and then incubated for 48 h. After 48 h, acetate or formate were added into the apical side, and incubated for 24 h. The average of actual TER values of Caco-2 monolayer used in this experiment was 846 ± 96 Ω cm^2^. Data represent the average of two or three independent experiments carried out in triplicate. (B) Effects of acetate or *B*. *bifidum WU12* on mRNA expression of TJ protein genes. TJ protein gene expressions in Caco-2 cells treated with acetate or *B*. *bifidum* WU12 for 24 h was were determined by quantitative RT-PCR. Data represent the average of three independent experiments carried out in duplicate or triplicate. Statistical differences were calculated by *t*-test (*P < 0.05).

We determined whether acetate and formate might be involved in TJ barrier restoration of Caco-2 monolayers whose TER were decreased approximately 20% by basolateral TNF-*α* stimulation for 48–72 h. Interestingly, administration of 10 and 20 mmol/L acetate to the apical side to the TNF-*α* treated Caco-2 monolayers showed a marked restorative effect after 24 h incubation (*P* < 0.05) (Fig.9A and Table7). On the other hand, the restorative effect of formate was weaker than the effect of acetate. Furthermore, occludin mRNA expression were significantly enhanced by apical stimulation with acetate (20 mmol/L) as the case of administration of *B*. *bifidum* WU12 (Fig.9B). Occludin mRNA levels by acetate or *B*. *bifidum* WU12 were 1.28-fold and 1.49-fold, respectively (*P* < 0.05). On the other hand, apical stimulation with acetate had no significant effect on the gene expression of claudin-1 and ZO-1.

**Table 7:** The TER value measured at the experiment of 24 h apical treatment of Caco-2 monolayers with varying concentrations of acetate and formate to induce the TJ barrier restorative effects

Sample	Before experiment		TNF-*α* treated		24 h treatment	
	TER (Ω × cm^2^)	±SD	TER (Ω × cm^2^)	±SD	TER (Ω × cm^2^)	±SD
Blank	768.1	120.0	787.2	165.6	800.1	162.0
TNF-*α*	875.0	89.5	698.2	91.6	678.8	92.9
Formate 2.5 mmol/L	809.9	95.3	669.3	92.0	669.3	156.1
Formate 5 mmol/L	828.0	89.1	688.4	78.6	743.2	114.9
Acetate 10 mmol/L	822.0	126.3	688.4	109.1	858.2	181.6
Acetate 20 mmol/L	823.8	105.0	691.0	97.7	868.7	144.6

### Dependency of Bifidobacteria-induced TER restoration on the differentiation stage of Caco-2 cells

**Figure 10:**
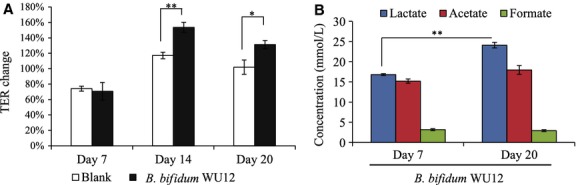
Characterization of *B*. *bifidum*-induced strengthening epithelial barrier function at different time points during Caco-2 monolayer polarization. (A) *B*. *bifidum*-induced TER enhancement: Day7, nonpolarization (actual TER values, 307 ± 77 Ω cm^2^); Day14, early-polarization (actual TER values, 508 ± 19 Ω cm^2^); Day20, stable-polarization (actual TER values, 806 ± 49 Ω cm^2^). TER changes were calculated from the TER value measured before and 24 h after the coincubation with *B*. *bifidum* WU12. Data represent the average of three independent experiments carried out in triplicate. (B) Short chain fatty acids production in nonpolarized (Day 7) or stable-polarized Caco-2 cells (Day 20) treated with *B*. *bifidum* WU12. Experiments were carried out in triplicate, and data represent the means ± SD. Statistical differences were calculated by *t*-test (**P < 0.01, *P < 0.05).

To investigate whether the TER-enhancing effect of *B*. *bifidum* depends on polarization of the intestinal epithelial cells, we determined the TER-enhancing activity in untreated Caco-2 cells at three different time points (Fig.10A and Table8): day 7, unpolarized; day 14, early-polarization; day 20, stable-polarization. In the early- and stable-polarization stages, the TER values of the Caco-2 cell monolayers significantly increased by approximately 30% in coculture with *B*. *bifidum* WU12. In contrast, *B*. *bifidum*-induced TER-enhancing effect in unpolarized Caco-2 cells was weak compared with that in polarized Caco-2 cells. Interestingly, the differential stage of Caco-2 cells did not affect the production of acetate and formate in the coculture of *B*. *bifidum* WU 12 and Caco-2 monolayers (Fig.10B). These results suggest that the acetate-induced TER-enhancing effect depends on the differentiation stage of the intestinal epithelial cells.

**Table 8:** The TER value measured at the characterization of *B*. *bifidum*-induced strengthening epithelial barrier function at different timepoints during Caco-2 monolayer polarization

Period	Sample	0 h		24 h coincubation	
		TER (Ω × cm^2^)	±SD	TER (Ω × cm^2^)	±SD
Day7	Blank	306.6	86.0	229.0	72.0
	*B*. *bifidum* WU12	307.5	94.3	224.1	94.8
Day 14	Blank	499.7	7.1	585.4	23.3
	*B*. *bifidum* WU12	516.9	6.2	794.5	38.8
Day 20	Blank	816.5	50.9	830.0	41.7
	*B*. *bifidum* WU12	796.1	35.6	1043.5	28.6

## Discussion

Dysregulation of intestinal permeability allows paracellular permeation of luminal antigens that initiate or promote intestinal inflammation. Thus, a defective intestinal TJ barrier has been proposed as an etiological factor for various gastrointestinal and systemic diseases with chronic inflammation, including allergy, celiac disease, Crohn's disease, diabetes, multiple sclerosis, and irritable bowel syndrome (Arrieta et al. 2006; Groschwitz and Hogan 2009). Recent studies demonstrated that various dietary components or probiotics regulate epithelial permeability by modifying intracellular signal transduction involved in the expression and localization of TJ proteins (Ulluwishewa et al. 2011). Administration of probiotics capable of colonizing the intestine is expected to induce long-term beneficial effects on intestinal health, whereas orally delivered dietary components have only a transient effect. In this study, we investigated the regulation of epithelial TJ barrier function by human fecal bacterial strains from the three bacterial genera (i.e., *Bifidobacterium*, *Enterococcus,* and *Lactobacillus*) most commonly used as probiotics for human consumption. This study demonstrated that some *Bifidobacterium* strains protect the epithelial TJ barrier against TNF-*α*-induced injury and promote the restoration of TNF-*α*-induced loss of epithelial barrier integrity. Furthermore, we showed that *B*. *bifidum*-induced restoration of epithelial TJ barrier may be attributed to increased production of acetate and formate, as demonstrated in cocultures of *B*. *bifidum* and Caco-2 cells.

Our present data showed that the permeability of Caco-2 epithelial cell monolayers exposed to live bacterial cells was altered depending on the bacterial strain. Many strains of *Bifidobacterium* species and some strains of *Lactobacillus* species significantly increased the TER of the Caco-2 cell monolayers, whereas all strains of *Enterococcus* species tested in this study dramatically decreased the TER. Therefore, in this study, we did not evaluate whether *Enterococcus* species have the capacity for the protection or restoration of the epithelial TJ barrier. However, these results do not mean that all of *Enterococcus* species are incapable of strengthening epithelial TJ function. Compared with probiotic strains belonging to the genera *Bifidobacterium* and *Lactobacillus*, *Enterococcus* species are used as probiotics to a much lesser extent because some strains possess virulence factors (including adhesins, invasins, pili, and hemolysin) and are resistant to various antibiotics (Franz et al. 2011). Steck et al. showed that a strain of the gut commensal *E*. *faecalis* compromises the epithelial barrier by metalloprotease-triggered degradation of the extracellular domain of E-cadherin (Steck et al. 2011). In contrast, some probiotic strains of *E*. *faecium* and *E*. *faecalis* are produced in the form of pharmaceutical preparations for treatment of diarrhea, immune modulation and lowering of serum cholesterol. Furthermore, Miyauchi et al. showed that pretreatment of heat-killed *Enterococcus hirae* prevented TNF-*α*-induced barrier impairment by modulating intracellular signaling pathways, suggesting that its cell wall components lead to the enhancement of the epithelial TJ barrier (Miyauchi et al. 2008). However, in this study, the heat-killed *B*. *bifidum* WU12 lost the TJ repair capacity completely. This suggests that the main inducing factor of this beneficial effect might be the metabolites of bacteria, not the cell wall components.

We found that different species of *Bifidobacterium* and *Lactobacillus* differentially attenuated TNF-*α*-induced reduction in TER of Caco-2 cell monolayers. Similarly, previous studies have shown that pretreatment with some commensal and probiotic bacteria can inhibit the increase in the epithelial TJ permeability caused by infection, proinflammatory cytokines, and stress (Resta-Lenert and Barrett 2003; Donato et al. 2010; Miyauchi et al. 2012). Molecular mechanisms underlying the protective effect are only partially understood. It is well established that treatment of intestinal epithelial cells with TNF-*α* promotes the redistribution of several TJ proteins (including claudin-1, occludin, and ZO-1) and impairs epithelial barrier function (Groschwitz and Hogan 2009). In particular, myosin light chain kinase (MLCK)-mediated phosphorylation of the myosin light chain (MLC), which induces the contraction of perijunctional actin–myosin filaments and opening of the epithelial TJ structure, appears to be a central molecular mechanism of TNF-*α*-induced loss of epithelial barrier function (Turner et al. 1997; Zolotarevsky et al. 2002; Ma et al. 2005; Shen et al. 2006). Previous studies showed that certain probiotic and commensal bacteria (i.e., *Enterococcus hirae* and *Lactobacillus rhamnosus*) suppressed the TNF-*α*-induced decrease in TER of Caco-2 cell monolayers by decreasing MLCK expression (Miyauchi et al. 2008, 2009). Furthermore, Ye et al. revealed that the TNF-*α*-induced increase in MLCK expression was mediated through activation of the nuclear transcription factor NF-*κ*B, which is considered as the master regulator of inflammatory responses (Ye et al. 2006). Thus, the suppression of TNF-*α*-induced NF-*κ*B activation, rather than modulation of TJ proteins, is thought to be a pivotal process for prevention of TNF-*α*-induced barrier impairment by commensal and probiotic bacteria. In other words, beneficial bacteria that confer preventive effects against TNF-*α*-induced TJ disruption could also exert antiinflammatory effects on intestinal epithelial cells by inhibiting NF-*κ*B activation.

There are only a limited number of studies focusing on the therapeutic effects of probiotics on impaired epithelial barrier function, as compared with multiple reports regarding preventive or protective effects of probiotics on TJ integrity against harmful stimuli (e.g., cytokines, infection, and H_2_O_2_). In a previous report, the probiotic *E*. *coli* Nissle 1917 led to restoration of a disrupted epithelial barrier of T84 cell monolayer caused by pathogenic *E*. *coli* infection, which was associated with increased expression and relocalization of zonula occludens-2 (ZO-2) (Zyrek et al. 2007). Our study demonstrated that *Bifidobacterium* species promoted the restoration of the epithelial TJ barrier of Caco-2 cell monolayer, in which TJ barrier integrity was impaired by pretreatment of TNF-*α*. Notably; *B*. *bifidum* WU12 exerted the highest TER-restorative effect on Caco-2 cell monolayers among the tested *Bifidobacterium* strains. Interestingly, *B*. *bifidum*-induced increase in TER was accompanied by an increase in mRNA expression of occludin. Balda et al. showed that the overexpression of occludin was linked to an increase in TER (Balda et al. 1996). From these findings, increased occludin gene expression may contribute to the ability of *B*. *bifidum* to restore the epithelial TJ barrier. In contrast, *B*. *bifidum* did not alter the mRNA expression of other TJ-related proteins (i.e., claudin-1 and ZO-1). In previous studies, the treatment of epithelial monolayers with probiotics (specifically *L*. *rhamnosus* GG, *L*. *plantarum,* and *L*. *salivarius*) enhanced protein expression of the occludin-associated plaque proteins (ZO-1, ZO-2, and cingulin) and other TJ-related proteins (claudin-1, claudin-3, and JAM-1) by IECs (Anderson et al. 2010; Miyauchi et al. 2012; Patel et al. 2012). Thus, epithelial barrier integrity may be further enhanced by administration of a mixture of multiple probiotic species each capable of modulating specific TJ proteins. Furthermore, the paracellular permeability of IECs are affected by expression levels and localization of TJ proteins in the paracellular space. Further study assaying the expression level and localization of TJ proteins during the *B*. *bifidum*-induced TJ restoration will be carried out to elucidate the cellular mechanisms.

Live *B*. *bifidum* had a restorative effect on the TER in TJ-impaired Caco-2 cell monolayers, whereas this effect was diminished when treated with heat-killed bacteria. It was previously reported that metabolites from *B*. *infantis* or *B*. *lactis* prevented epithelial barrier dysfunction caused by proinflammatory cytokines or pathogen infection (Ewaschuk et al. 2008; Putaala et al. 2008). Thus, we speculate that some of *B*. *bifidum*-derived metabolites may also regulate the epithelial TJ. In this study, NMR-based metabonomics analysis of the coculture supernatant revealed that SCFAs (acetate and formate) are responsible for the TER-restorative effect induced by live *B*. *bifidum*. Interestingly, production of acetate and formate was upregulated in the coculture environment compared with the monoculture of *Bifidobacterium* strain or Caco-2 monolayers, indicating that cross talk between the host and microbes may alter their metabolic activity. Further studies are needed to clarify which cells enhanced the production of acetate or formate by coculture. Furthermore, we observed that production levels of acetate and formate in the coculture varied depending on the *Bifidobacterium* strain. There are previous reports showing the beneficial effect of SCFAs on TER and paracellular permeation in Caco-2 cell monolayers (Mariadason et al. 1997; Malago et al. 2003; Suzuki et al. 2008; Elamin et al. 2013). Notably, Suzuki et al. presented the finding that the incubation of Caco-2 cell monolayers with acetate (>40 mmol/L) led to a significant increase in the epithelial TJ barrier function. They suggested that acetate-induced TJ enforcement may be mediated by intracellular signal transduction through GPR43, which has been defined as one of the cell surface G-protein coupled receptors for SCFAs (Brown et al. 2003; Suzuki et al. 2008). However, the molecular mechanism underlying acetate-mediated fortification of intestinal epithelial barrier remains unclear. In contrast, our data suggest that *B*. *bifidum*-induced epithelial TJ enforcement was dependent on the differentiation stage of Caco-2 cell monolayers. In future experiments we will elucidate the molecular mechanism involved, including the investigation of the GPR43-acetate signaling pathway.

Probiotic microorganisms (e.g., *Bifidobacterium* and *Lactobacillus*) exert their immune-modulatory effect through interaction with Toll-like receptor 2 (TLR2), which recognizes cell wall components such as peptidoglycan, lipoteichoic acid, and lipoprotein (Mohamadzadeh et al. 2005; Galdeano and Perdigon 2006; Hoarau et al. 2006; Zeuthen et al. 2008). Furthermore, recent studies have shown that TLR2 stimulation plays a crucial role in maintaining intestinal epithelial barrier integrity. Stimulation with the synthetic TLR2 agonist PCSK efficiently preserves TJ-associated barrier integrity (Cario et al. 2004, 2007). Cell wall components derived from some *Bifidobacterium* and *Enterococcus* strains are also reported to markedly enhance epithelial TJ barrier through a TLR2-mediated mechanism (Miyauchi et al. 2008; Sultana et al. 2013). This study showed that heat-killed *B*. *bifidum* slightly increased TER of Caco-2 cell monolayers, whereas lipase and mutanolysin treatment to digest lipid-related components abolished the rise in TER elicited by *B*. *bifidum*. These results suggest that cross talk between *B*. *bifidum* and TLR2 plays a partial role in the modulation of intestinal epithelial TJ barrier function.

In summary, we demonstrated that most of *Bifidobacterium* strains have the capacity to prevent TNF-*α*-induced disruption of intestinal epithelial barrier and to promote epithelial TJ integrity. Furthermore, it was found that the upregulation of the production of SCFAs (acetate and formate) leads to the restoration of the epithelial TJ barrier. Our findings show that TJ-strengthening *Bifidobacterium* strains could make an important contribution to the prevention and treatment of various digestive system disorders associated with intestinal epithelial barrier dysfunction (including diabetes, inflammatory bowel disease, and nonalcoholic fatty liver disease). However, studies using animal models are still required to evaluate in vivo the beneficial effects seen here in vitro.

## Conflict of Interest

The authors have no conflicts of interest to disclose.
